# Modeling the Depth of Surface Cracks in Brake Disc

**DOI:** 10.3390/ma14143890

**Published:** 2021-07-12

**Authors:** Wojciech Sawczuk, Mateusz Jüngst, Dariusz Ulbrich, Jakub Kowalczyk

**Affiliations:** 1 Department of Transport and Civil Engineering, Institute of Transport, Poznan University of Technology, 60-965 Poznan, Poland; wojciech.sawczuk@put.poznan.pl (W.S.); mateusz.jungst@put.poznan.pl (M.J.); 2 Department of Transport and Civil Engineering, Institute of Machines and Motor Vehicles, Poznan University of Technology, 60-965 Poznan, Poland; jakub.kowalczyk@put.poznan.pl

**Keywords:** brake disc, surface cracks, railway disc brake

## Abstract

The article presents the state of knowledge and research in the field of surface cracks occurring in disc braking systems of rail and car vehicles. The craze formed during the operation of vehicles is particularly dangerous and leads to breaking the disc into several pieces. It may lead to a loss of braking force and damage to the entire disc brake caliper. The main aim of the research is to identify surface cracks in brake discs made of cast iron and use experimental methods to estimate their depth. Research were conducted on the disc braking system developed by the authors. In examining the location and depth of cracks, the penetration method, ultrasound, as well as a special probe were used. This device measures the crack depth based on the electrical resistance between two points on the surface of the metallic object. The tests showed that the first microcracks on the brake discs appeared after 309 braking tests on the test stand. In addition, it was observed that the surface cracks length of the disc increased linearly to depth until they reached about 11.5–12 mm with corresponded to crack lengths in the range of 65–70 mm. However, determination of the regression functions presented in the article allows to estimate the depth of surface cracks up to 70 mm long on cast iron brake discs by measuring their length.

## 1. Introduction

Most of the publications taking thermal phenomena in brake discs into consideration focus on the destructive effects of friction rings on the disc material. It has been shown that changes in the volume of the material due to its thermal expansion in the areas of overheating lead to compressive stresses during heating and tensile stresses during cooling. Multiple repetitions of this cycle lead to a gradual increase in stresses up to the limit of the tensile strength of the disc material. This phenomenon, described in many publications [1,2,3,4,5,6,7,8], causes cracks that grow with each successive braking of the vehicle.

**Figure 1:**
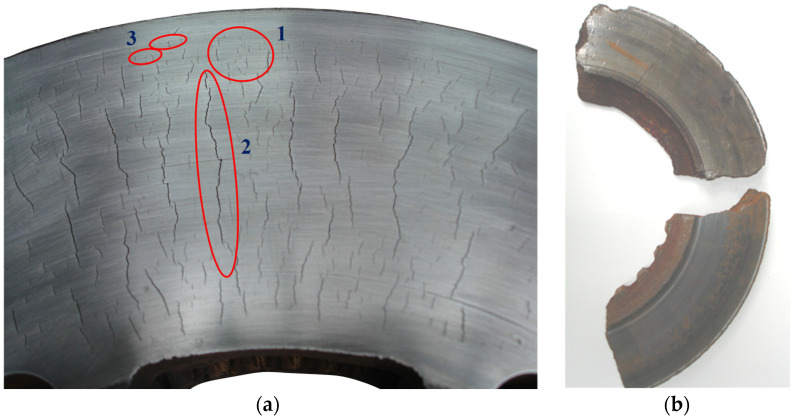
View of a damaged brake disc: (**a**) cracks on the friction surface of the disc; 1—microcracks, 2—radial cracks, 3—circumferential (longitudinal) cracks, (**b**) view of fragments of a cracked brake disc.

In [9], three basic surface cracks were distinguished, caused by thermal and mechanical stresses in the friction ring of the disc. These were microcracks, radial cracks and circumferential cracks, which the authors of this article also observed during their own observations and research. However, circumferential cracks are rare and circumferential scratches are more common. Circumferential cracks are caused by the penetration of hard debris between the cladding and the disc, such as sand grains from a track or road. Scratches are not related to thermo-mechanical phenomena occurring during braking. The number of circumferential cracks in long-term operation changes due to the phenomenon of abrasive friction. Some of the scratches are ground in subsequent braking and new ones appear. A view of a brake disc with various failures and an example of the disc fragments after its fracture are shown in Figure 1.

**Figure 2:**
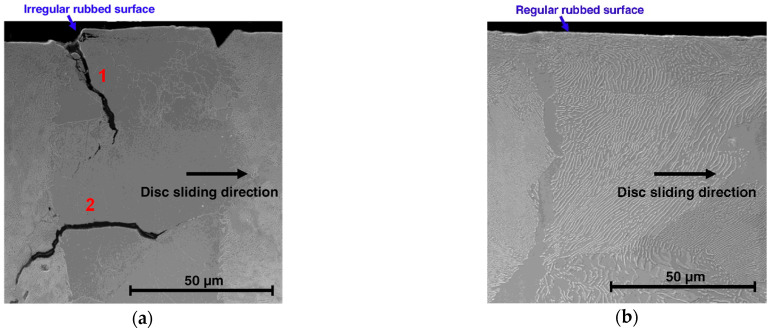
Images of the cross-sections of the friction ring of the brake disc obtained with a scanning electron microscope [10]: (**a**) cross-section through the area under the macroscopic hot-spot: (1) fracture perpendicular to the friction surface and (2) fracture parallel to the friction surface; (**b**) cross-section through the area free from overheating.

Radial cracks usually begin inside the material and on the rim of the recorded macroscopic hot spots [10]. In addition, radial cracks (Figure 2a) grow from a single fracture or by the joining of successive fractures along the same radius (the most frequent phenomenon), which has been confirmed by researchers [11,12].

Moreover, [10] showed that, as a result of the solid-solid phase transformations and mechanical stress, cracks can also occur parallel to the friction surface in the depth of the material. This is a phenomenon that contributes to the spalling of a fragment of the brake disc. The abovementioned study also confirmed that the areas where no local or macroscopic overheating occurred are free from mechanical damage; then, there are no cracks on the friction surface (Figure 2b).

**Figure 3:**
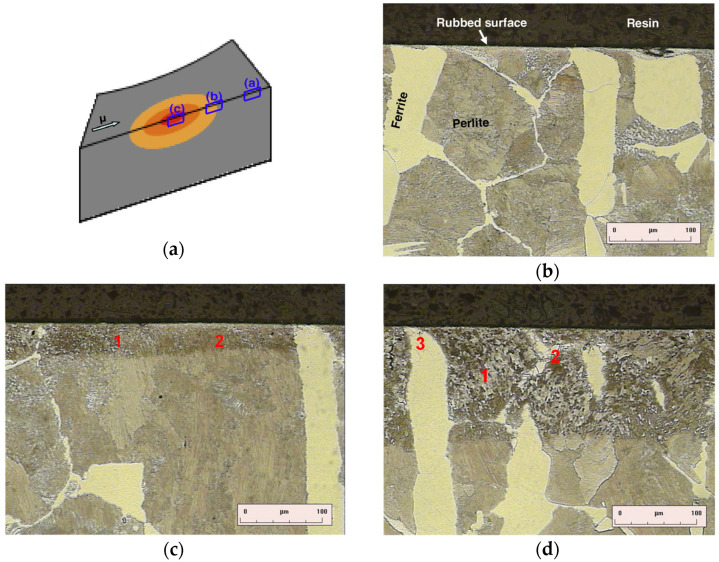
Images of the structure obtained with an optical microscope: (**a**) model of the target section with measurement points [10], (**b**) cross-section outside the overheating area [10], (**c**) cross-section on the edge of the overheating area [10], (**d**) cross-section directly in the overheating area: 1—bainite, 2—fine-grained perlite, 3—fine-grained ferrite [10].

Observations of phase transformations in the material structure indicated that local overheating causes changes in the structure of the disc to a depth of about 120 µm [13]. Tests on a disc made of carbon steel C48 for surface hardening proved the presence of only two components of the phases, i.e., the α_f_-ferrite phase containing less than 0.02% carbon and eutectic perlite containing about 0.8% carbon. Both phases, due to overheating, transform into the γ-austenite phase. The distribution of austenite precipitates depends on the cross-section of the material due to different austenitizing temperatures of the primary phases from 890 to 720 °C. Then, as a result of cooling, the austenitized areas proceed in a reverse transformation, where ferrite appears in the form of a finer grain and the perlite occurs in the form of a mixture of bainite and fine pearlite, as shown in Figure 3.

In order to avoid the phenomenon of surface cracks on brake discs, many research centers have conduct research on new materials for brake discs described in the articles [14,15,16,17,18,19]. These works are preceded by various numerical analyses in terms of stress concentrations, which were also presented in [20,21,22,23,24,25,26,27,28].

Knowledge of the fracture mechanisms and the geometrical parameters of the fractures is crucial from the point of view of the operation of vehicles equipped with disc brakes. Thermal cracks in the friction surface usually cause disc replacement with new ones, much earlier than it would be necessary to wear the material. As long as the cracks are shallow, they can be removed by turning the disc. Nevertheless, if their depth is greater than the manufacturer’s recommended maximum thickness loss of the friction ring, this operation will not make sense, because the regenerated disc will not be allowed for further use and will need to be replaced with a new one. This issue has not been analyzed so far, which created an unfilled gap in the practical application of previously conducted research around the world. In this article, a simple tool that will allow, based on simple measurements, to determine whether a brake disc can be regenerated and still be in service was created. Thus, this tool will allow to reduce the number of unnecessary replacements of this component and the operating costs of the vehicle.

The main goal of the research is to identify the surface cracks of rail vehicle brake discs and to develop a method for estimating the depth of the crack on the basis of its length. Determining both the crack length, as well as depth, is essential from the point of view of the safety of vehicles with disc brakes. The research used mainly non-destructive methods, and the developed models of the crack depth will allow better and faster diagnostics of brake disc cracks in the future.

## 2. Methodology of Testing Surface Cracks

Research in the field of identifying cracks on brake disc surfaces was carried out in several stages. First, bench tests were carried out on railway brake discs. This research was conducted using the same methodology as in [29,30]. The research was carried out in at Łukasiewicz Research Network IPS TABOR in Poznan, Poland. During these tests, two brake discs, T1 (Nodular Cast Iron) and T2 (Grey Cast Iron), with dimensions of 610 mm × 110 mm (diameter × disc thickness) were used. The main difference between the discs was the chemical composition. The T2 disc had a higher copper content than the T1 disc. The discs were prepared for testing in accordance with [31,32]. The program for the stand tests is presented in Table 1. The tests were carried out taking into account the pad test procedure contained in [33].

In the second part of research, penetration tests were performed to observe the actual number of surface cracks and their length. The research was based on the following publications [34,35]. In the third part of the research, tests were carried out to assess the depth of surface cracks.

Due to the nature of surface cracks and the shape of the brake disc, it is currently difficult to investigate the depth of this defect using known non-destructive methods (such as ultrasonic, magnetic or other methods). In such types of cracks, it is best to use crack depth gauges with a potential probe, i.e., the RMG 4015 instrument (Karl Deutsch GmbH, Wuppertal, Germany). Determining the depth of cracks with a potential probe according to [36] is based on measuring the electrical resistance between two points on the surface of the metallic test object. When there is a crack between these two points, the electrical resistance is greater than for a non-cracked surface. With alternating current, the electric field lines are displaced outwards from the material in the area below the surface. With increasing frequency, the current density also increases as does the penetration depth, δ_p_, after which the current density decreases by 63%, and is shown by the relationship [37]:

(1) $$\delta_{p}=\frac{1}{\sqrt{f_{p}\times \pi \times \mu_{o}\times \sigma_{e}}}$$

where:

σ_e_—specific electrical conductivity, S/m;

μ—relative magnetic permeability;

μ_o_—magnetic permeability constant, H/m;

f_p_—Alternating current frequency Hz.

**Figure 4:**
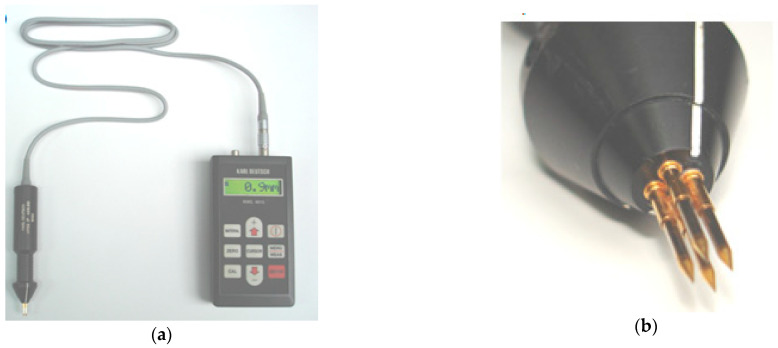
Instrument for crack depth measurement, type RMG 4015, made by Karl Deutsch: (**a**) general view and (**b**) head for cracks perpendicular to the surface.

Figure 4 shows a surface crack depth Karl Deutsch measuring instrument (Karl Deutsch GmbH, Wuppertal, Germany), with a perpendicular cracking head.

The probe shown in Figure 4b is four-pole in a square configuration and consists of two current and two measurement poles. The tips of the probe in relation to the pilot sleeves are elastically mounted and made of hardened steel, which ensures good electrical contact with the test object. In addition, the measuring probes are also available in a four-pole system with poles arranged in series to measure cracks occurring at an angle to the surface (plane) of the object.

## 3. Penetrant Test Results

**Figure 5:**
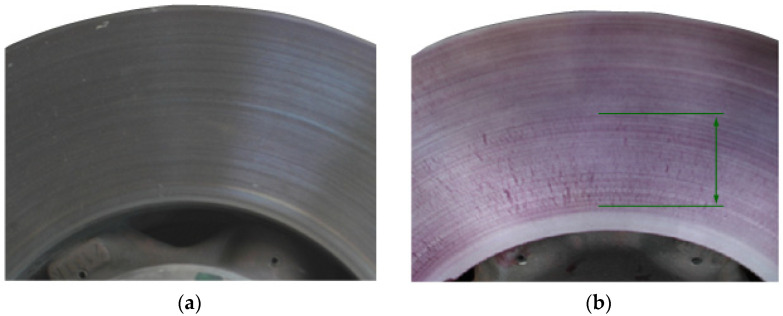
Condition of the friction surface of the T1 (Nodular Cast Iron) disc after the first braking: (**a**) before penetration testing and (**b**) after penetration testing (place of cracks marked green).

**Figure 6:**
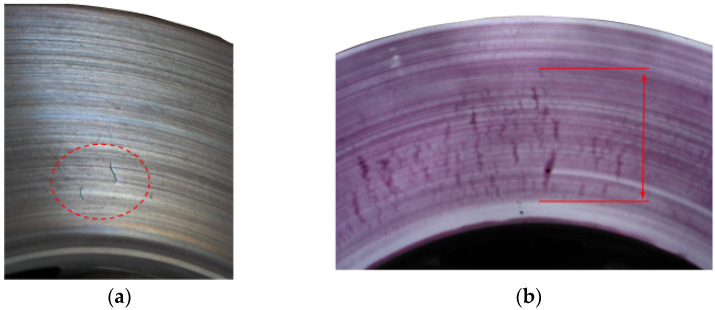
T2 (Grey Cast Iron) disc surface condition after successive braking (places of cracks marked red): (**a**) before penetration testing and (**b**) after penetration testing.

Bench tests of brake discs after 309 brakings, combined with non-destructive (penetration) tests, showed that surface cracks appeared on new discs after several hundred braking operations. The first microcracks in the area of the inner diameter of the disc are visible only with the use of penetration tests, as shown in Figure 5. Further braking on the test bench disclosed the first cracks (3 cracks) with a length not exceeding 7 mm, visible to the eye, and are shown in Figure 5 and Figure 6.

The brake discs were tested according to the schedule presented in Table 1. The appearance of the first surface cracks on the T2 disc was related to the grade of cast iron and the composition of the chemical elements (additives). In the case of the disc T2, gray cast iron was used, while disc 1 was made of nodular cast iron. The chemical compositions as a percentage of additives are presented in Table 2. The discs were not subjected to thermo-chemical treatment.

The appearance of the first surface cracks does not disqualify the brake disc from further use. Only when the cracks grow to a dangerous size across the entire width of the ring it is necessary to replace the disc or regenerate of the friction surface [38].

## 4. Examination of the Depth of Surface Cracks

Surface cracks occurring in brake discs during repeated braking as a result of exceeding the crack length in accordance with the technical and operational documentation of rail vehicles [39,40] force the vehicle owner to regenerate the friction surface or even replace the disc. Although the abrasive wear measured on the thickness of the disc is not exceeded, due to thickness of the brake disc being about 20–25 mm, it is important to determine the depth of cracks in order to check if a crack is not through the entire disc. In each case, it was found that, in rail vehicles, brake discs are dismounted due to too many individual surface cracks, although there was no evidence of a linear wear increase in the thickness of the disc as a result of friction between the pads and the brake disc. In some cases, the friction surfaces of the discs are machined on a lathe. However, if surface cracks are still visible after turning, the disc should be replaced with a new one. The portable device used by the authors is the RMG 4015 device made by Karl Deutsch (Wuppertal, Germany). In the research presented in this article, the indication of crack depth measured with the RMG 4015 device was checked, as well as the actual depth of the crack. In addition, the ultrasonic method was used because it allows the location of cracks and the assessment of the condition of machines and individual joints [41,42]. In addition, an ultrasonic magneto-particle test (Karl Deutsch GmbH, Wuppertal, Germany) was performed.

**Figure 7:**
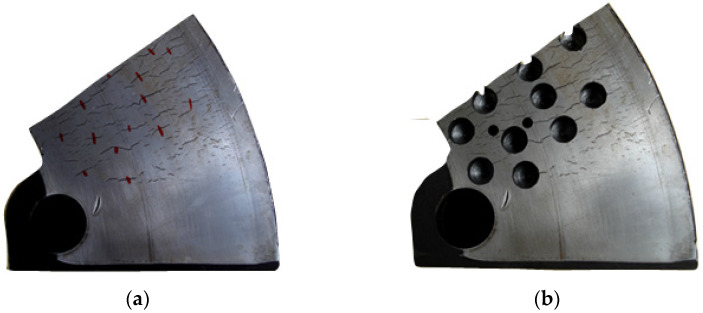
View of the 610 mm × 110 mm brake disc segment: (**a**) with the red half-crack line marked, (**b**) with blind holes to observe the crack depth.

For the friction ring segment of the 610 mm × 110 mm-type brake disc, holes were made in several microcracks in the middle of their lengths, which is shown in Figure 7. Thanks to this, it was possible to observe the actual crack depth and the crack shape. The cracks shown in Figure 7a were measured prior to making the blind holes.

**Figure 8:**
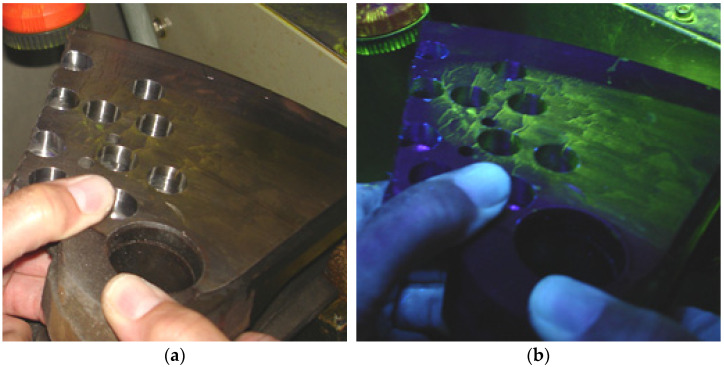
View of the brake disc section 610 mm × 110 mm: (**a**) before magneto-particle tests, (**b**) during tests.

Additionally, in order to better observe the shape of the crack cross-section and its depth, magneto-particle tests were performed and the result are shown in Figure 8.

**Figure 9:**
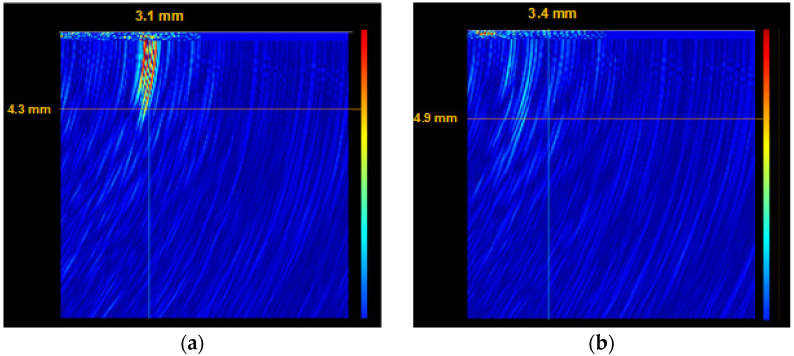
View of the cross-section of selected microcracks of the brake disc section obtained with the GEKON ultrasonic flaw detector: (**a**) crack with a depth of 4.3 mm and (**b**) crack with a depth of 4.9 mm.

Then, the cracks indicated in Figure 7a were examined using the ultrasonic method with a GEKON (Karl Deutsch GmbH, Wuppertal, Germany) ultrasonic flaw detector to assess the depth of the crack. Figure 9 shows two selected visualizations of the crack depth.

On 14 marked microcracks on the brake disc section (Figure 7a), and the crack depths measured with the RMG 4015 device, as well as those verified with the GEKON ultrasonic flaw detector, the relative percentage error of the measurement with the RMG 4015 device was in the range of 9–16%. Taking this into account, it was concluded that the RMG 4015 device can be used in measuring the depth of cracks at the surface of brake discs of rail vehicles.

**Figure 10:**
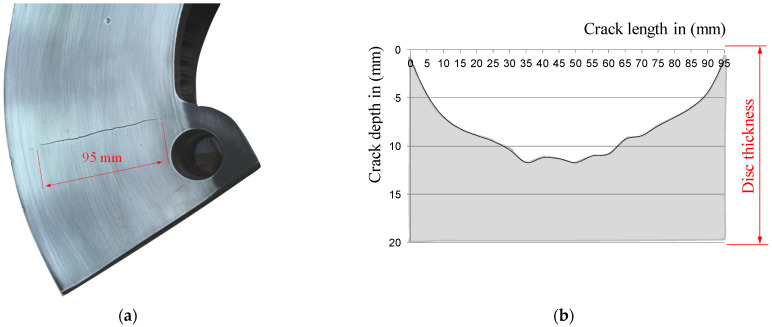
One-sided crack on the ring of the railway brake disc: (**a**) view of the crack and (**b**) the relationship between the length of the crack and its depth, measured with the RMG 4015 device.

**Figure 11:**
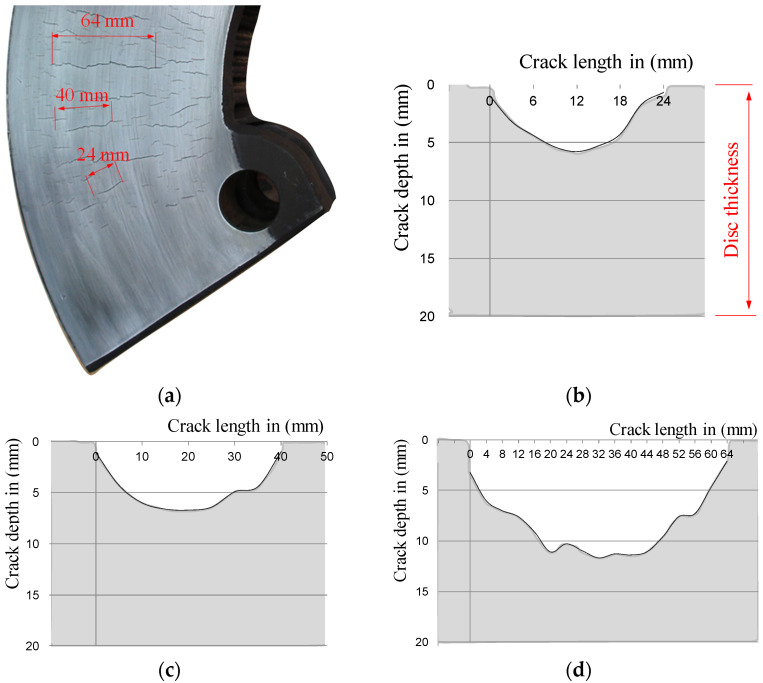
Surface cracks (microcracks) on the ring of the railway brake disc: (**a**) microcracks mesh on the BK141 segment of the disc, (**b**) disc cross-section with a 24 mm long crack, (**c**) disc cross-section with a 40 mm long crack, and (**d**) disc cross-section and a crack with a length of 64 mm.

Then, the depth of surface cracks was measured on subsequent parts of the brake discs, every 5 mm of the crack length. Figure 10 and Figure 11 show the results of testing the depths of selected surface cracks using the potential probe of the RMG 4015 instrument, along with the view of these cracks.

For further research, of which the main goal was to determine the depth of surface cracks, three brake discs were selected randomly from passenger carriages running in the planned national timetable. Due to numerous microcracks, the length of which exceeded the requirements set out in the regulations [39,40] regarding the maintenance of these wagons, the discs were dismantled after a period of about five years. The number of microcracks on one side of the disc ranged from 40 to 60, depending on the tested disc. Then the surface cracks were grouped into classes of their length intervals, every 5 mm, in order to determine the ratio of the microcrack length to its depth.

**Figure 12:**
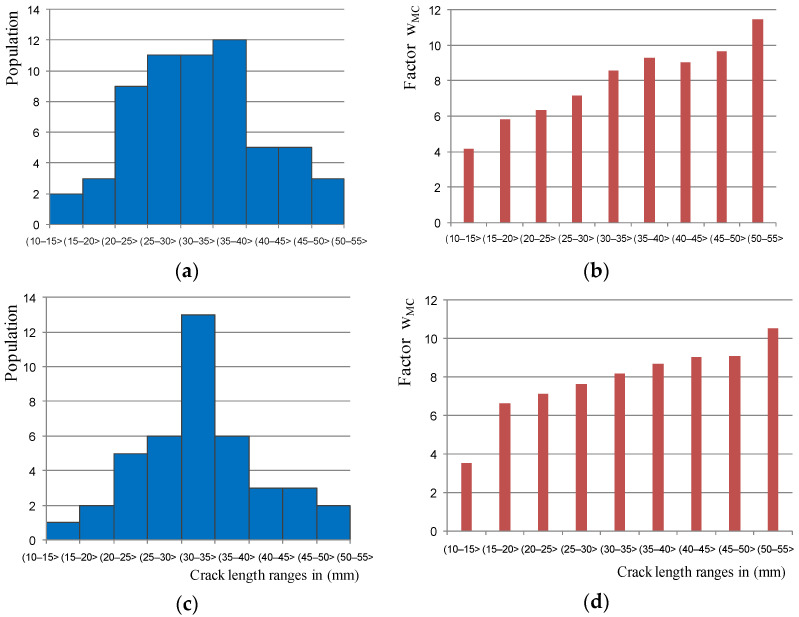
Results of surface crack depth measurements for selected brake discs: (**a**) crack number histogram for the first disc, (**b**) dependence of the w_MC_ coefficient (crack length to its depth) for individual classes of crack length intervals of the first disc, (**c**) crack number histogram for the second disc, and (**d**) w_MC_ relationship for the second target.

Figure 12a,c shows histograms of the number of thermal cracks on one side of the brake disc. In order to determine the relation of the crack length to its depth the coefficient w_MC_ was introduced as the relationship l_p_/g_p_, as shown in Figure 12b,d for various crack length ranges. The w_MC_ coefficient was determined as the quotient of the crack length in a straight line to the greatest crack depth recorded with the RMG 4015 Karl Deutsch device.

Analyses of all the cases of the tested brake discs allowed to determine that the largest group of microcracks were cracks with a length of 30 to 40 mm. Only in a few cases were cracks longer than 55 mm observed. Moreover, it was found that in each case of the tested brake discs, the microcrack l_p_/g_p_ coefficient increased logarithmically with the increase in the crack length, which was evidenced by the high coefficient of determination above 0.9 for this function in relation to other approximating functions. It should be noted that Figure 12 shows the results of only two brake discs. In the main tests for measuring the depth of surface cracks and proving that there is a dependence of the depth to the length of the crack, a total of five different brake discs made of gray and nodular cast iron were tested. The brake discs came from various rail vehicles, such as passenger carriages and rail buses. The tested discs had the following number of cracks on both sides: 124 (T3 disc), 94 (T4 disc), 82 (T5 disc), 80 (T6 disc), and 103 (T7 disc). In the research related to the measurement of the depth of surface cracks, 492 measurements were made, including the fragment of the disk shown in Figure 7. These measurements were used for further modeling in terms of the depth and shape of the crack. Table 3 presents the ranges of the l_p_/g_p_ coefficient for the tested brake discs.

Based on the data of the range of surface crack lengths and the range of w_MC_ coefficients, the relationships for the estimation of the minimum (l_p_, min) and maximum (g_p_, max) brake disc crack depth were determined:

(2) $$g_{p,\;\min}=\frac{l_{p}}{4.2};\;g_{p,\;\max}=\frac{l_{p}}{3.5};\;\;l_{p}\in (10-15\rangle \;\mathrm{mm}\;g_{p,\;\min}=\frac{l_{p}}{6.6};\;\;g_{p,\;\max}=\frac{l_{p}}{5.8};\;\;l_{p}\in (15-20\rangle \;\mathrm{mm}\;g_{p,\;\min}=\frac{l_{p}}{7.1};\;\;g_{p,\;\max}=\frac{l_{p}}{6.3};\;\;l_{p}\in (20-25\rangle \;\mathrm{mm}\;g_{p,\;\min}=\frac{l_{p}}{7.6};\;\;g_{p,\;\max}=\frac{l_{p}}{7.2};\;\;l_{p}\in (25-30\rangle \;\mathrm{mm}\;g_{p,\;\min}=\frac{l_{p}}{8.5};\;\;g_{p,\;\max}=\frac{l_{p}}{8.2};\;\;l_{p}\in (30-35\rangle \;\mathrm{mm}\;g_{p,\;\min}=\frac{l_{p}}{9.1};\;\;g_{p,\;\max}=\frac{l_{p}}{9.0};\;l_{p}\in (35-40\rangle \;\mathrm{mm}\;g_{p,\;\min}=\frac{l_{p}}{9.2};\;\;g_{p,\;\max}=\frac{l_{p}}{9.0};\;l_{p}\in (40-45\rangle \;\mathrm{mm}\;g_{p,\;\min}=\frac{l_{p}}{9.7};\;\;g_{p,\;\max}=\frac{l_{p}}{9.1};\;l_{p}\in (45-50\rangle \;\mathrm{mm}\;g_{p,\;\min}=\frac{l_{p}}{11.4};\;\;g_{p,\;\max}=\frac{l_{p}}{10.5};\;l_{p}\in (50-55\rangle \;\mathrm{mm}\;$$

Based on the above dependences, Equation (2), it was possible to determine the crack depth range on the brake disc, based on measurement of the microcrack length. This is especially useful and justified from the point of the maintenance process, where an employee who inspects the brake system does not have sufficient tools to measure the depth of a surface crack.

## 5. Modeling the Shape and Depth of a Surface Crack

**Figure 13:**
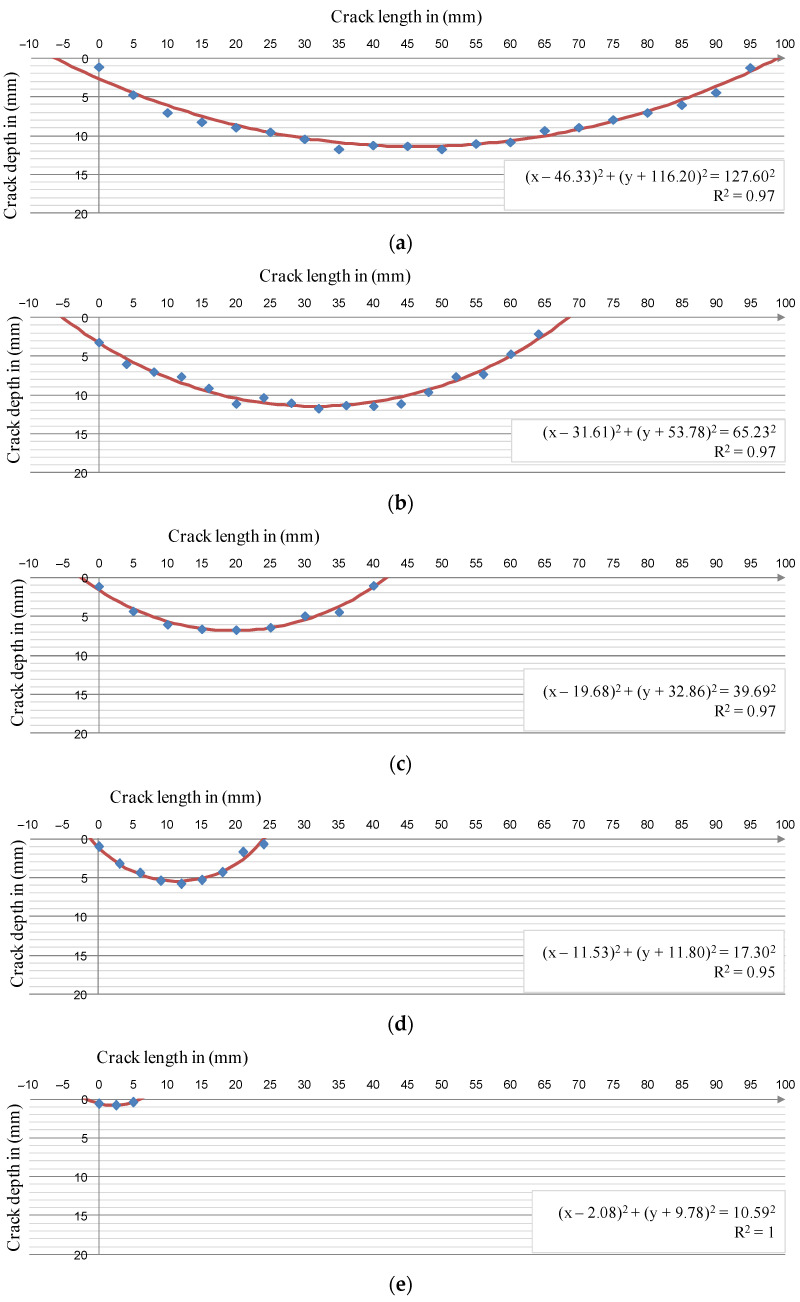
Crack depth courses depending on their length; blue—measurement results, red—results from the regression model: (**a**) crack 95 mm long, (**b**) crack 64 mm long, (**c**) crack 40 mm long, (**d**) crack 24 mm long, and (**e**) crack 5 mm long.

The result of research presented in the article has shown that the depth of a surface crack is strictly dependent on its length. The course of depth changes at successive measurement points along the crack has the shape of an arc of a circle. For this reason, for the performed measurements, an attempt was made to create regression functions of a circle (the curves are shown in Figure 13).

Obtained graphs and curves indicate a certain discrepancy at both ends of the crack between the regression function and the actual results—the theoretical crack is always a few millimeters longer than the real one. The reasons for this discrepancy were both in the principle of operation of the measuring probe and in the behavior of the target material itself; however, all regression functions showed a very good fit with the measurement results.

Based on the regressive functions of the circles, it was possible to model the radius of the circle depending on the crack length to determine the crack depth; however, the longest (95 mm-long) crack was not taken into account when the model was created. The observations made thus far show that the surface cracks of the disc increased their depth linearly until they reached about 11.5–12 mm (achieved the range of 65–70 mm of crack length). For longer surface cracks, their depth stabilizes approximately at a constant level. Moreover, based on the available documentation, cracks exceeding a length of 70 mm qualify a disc for decommissioning [37,38]. The relationship between the crack length and the radius defining its depth is described by Equation (3) [43]:

(3) $$r=0.968\times l_{p}+1.020$$

where:

r—crack profile defining radius in mm;

l_p_—crack length in mm.

The function determining the position of the center of the circle on the ordinate axis is described by the Equation (4):

(4) $$b=-0.795\times l_{p}-0.629$$

where *b*—coordinate of the center of the circle on the ordinate axis.

Adding up both of the above dependencies, the dependence on the crack depth (g_p_ in mm) depending on the crack length (l_p_) was obtained, which is described by Equation (5):

(5) $$g_{p}=r+b=0.173\times l_{p}+0.391$$

**Figure 14:**
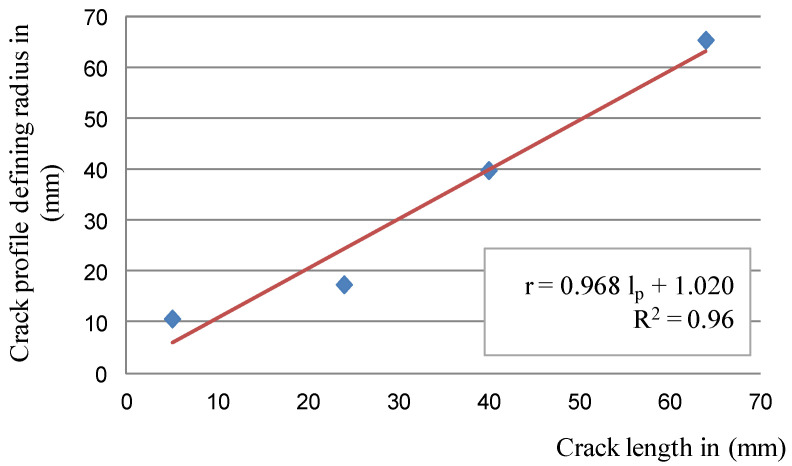
The relationship between the radius of the arc defining the crack depth and its length; blue—radius values for individual cracks determined from the regression functions of circles, red—linear regression for these values.

**Figure 15:**
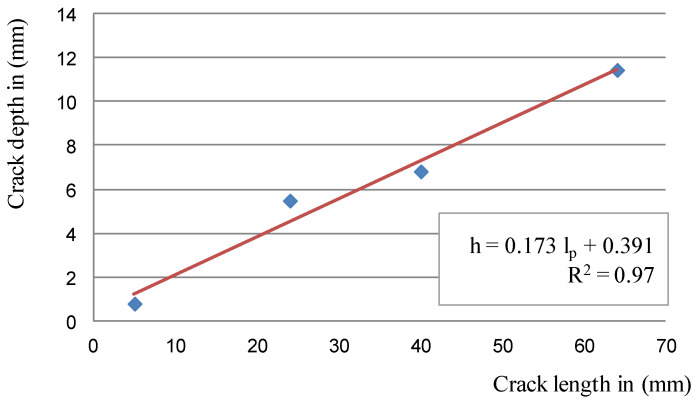
The dependence of the depth of the crack on its length; blue—depths determined from the regressive functions of circles, red—linear dependence of the crack depth on its length.

Dependencies (3) and (5) are shown in Figure 14 and Figure 15.

The designated regression functions for estimating the depth of the crack give high coefficients of determination R^2^ at the level of 0.95–0.97, which proves that the model is well-adjusted to the measurement results. Thus, it can be concluded that the depth of surface cracks, up to 70 mm long, on cast iron brake discs can be determined by simply measuring their lengths.

## 6. Discussion

**Figure 16:**
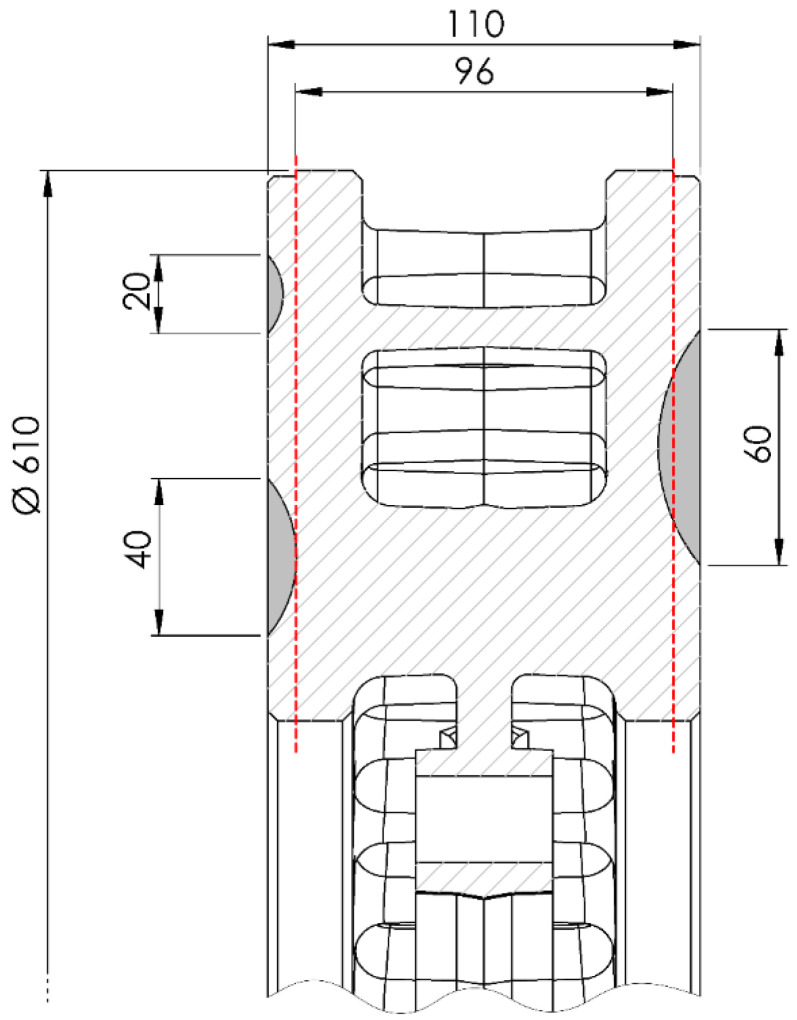
Visualization of three cracks with lengths of 20, 40 and 60 mm on the surface of the fragment of brake disc with dimensions of 610 mm × 110 mm; the red line marks the minimum thickness of the brake disc (96 mm).

Conducted research and analysis of the results allowed for the creation of a simple tool enabling an easy assessment of the technical condition of the brake disc in terms of its suitability for regeneration and further operation. Figure 16 shows examples of 20-, 40-, and 60-mm-long crack cross-sections in a brake disc, with a nominal size of 610 mm × 110 mm, the permissible reduction in the friction ring thickness is 7 mm on each side of the disc.

The longest of the cracks, according to the model, reaching a depth of 10.77 mm, excluded the disc from further use. Even if the disc is regenerated to the minimum thickness, the crack will not be completely eliminated. Shorter cracks, respectively, 3.85 and 7.31 mm in depth, provided that no more serious damage occurs in the remaining cross-sections of the friction rings, could be eliminated using appropriate surface treatment of the disc.

**Figure 17:**
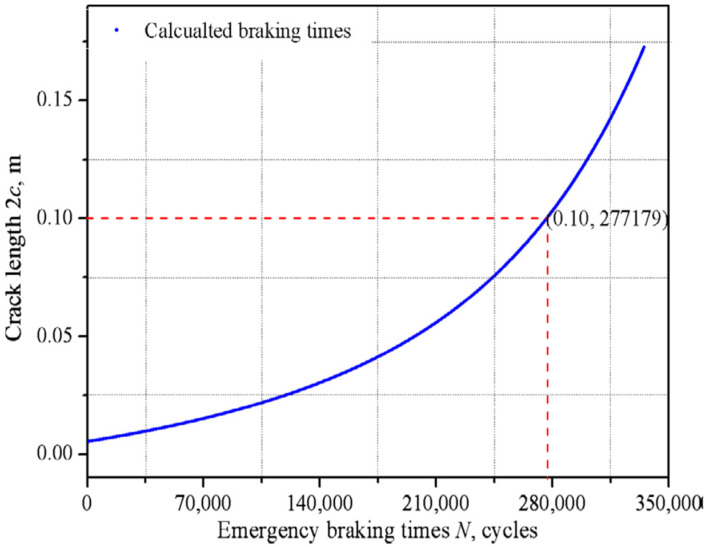
Expected crack length depending on the number of emergency braking cycles performed; the limit crack length of 100 mm was reached in this case after 277,179 braking cycles [8].

As indicated in [8], the increase in the crack length with each braking is the greater, the longer the crack is (Figure 17). Therefore, a reasonable solution would be to regularly turning the brake discs with a small repair dimension in order to eliminate still relatively short cracks; at most a few millimeters. Such a procedure would prevent the penetration of the fracture into the ring cavity due to the ever-faster propagation, thus extending the life time of the brake disc.

**Figure 18:**
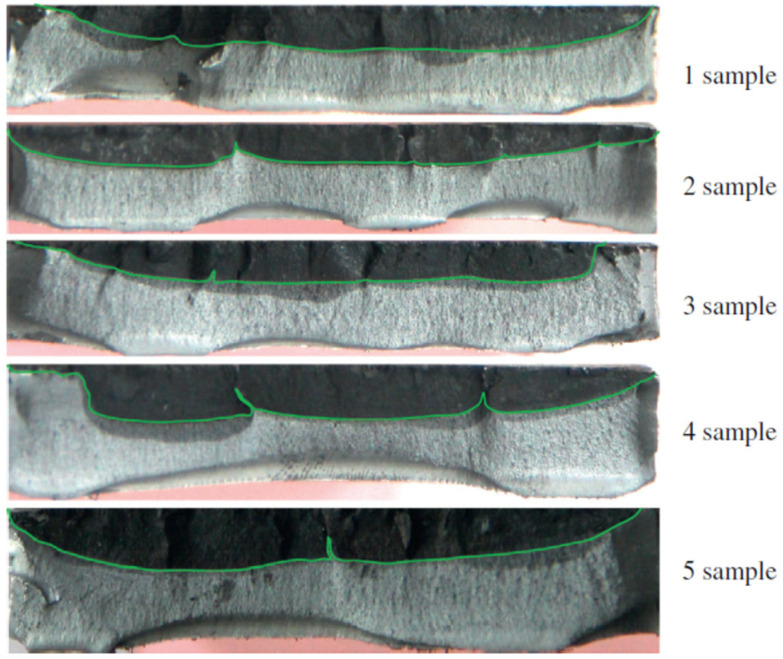
Macro morphologies of the crack fractures [12].

The brake disc material, or, more precisely, its grain, is also important for the method of generating a crack. Since cracks can propagate in two ways: through the growth of a single crack (less frequently) or by propagating and joining several cracks along the same radial axis (more often), due to the different elasticity of the materials used for the discs. The final depth of the crack can differ at different stages of the failure. Finally, a fully developed long crack will reach a similar depth regardless of the mechanism of its formation, and the only difference in the image of the crack will be point “artifacts” in the form of local flattening of the crack (Figure 18, samples 2–5) delineating the boundaries between primary cracks [12]. For this reason, the developed method of crack assessment is universal for practical applications. Moreover, it should be pointed out that the results of the crack shape obtained in the tests are consistent with the results of other studies [5,6,7,8,11,12].

The process of replacing brake discs mounted to the axle of a wheelset is very complex—in the case of monolithic discs, it requires pressing the wheel from the axle, and only then it is possible to dismantle the disc. Then, a new disc is mounted and the wheel is pressed on again. Since the wheel mounting must be as rigid as possible, performing this operation on a single set is possible only a few times before the axle mounting is subject to extreme wear—then, in fact, the entire wheel set qualifies for replacement. For this reason, minimizing the number of brake disc replacements is of interest to railway carriers and repair plants. Early elimination of the effects of thermal cracks will reduce the number of premature component replacements, and, thus, in the long term, will reduce the operating costs of rail vehicles.

## 7. Conclusions

The conducted tests showed that it is possible to determine the depth of a crack on the basis of their length. Cracks that do not disqualify the brake disc from use already at the stage of visual assessment (e.g., due to a crack in the ring along its entire thickness) can be assessed by measuring their length alone.

This article presents two methods of calculating the depth of surface cracks on the basis of a complex and multi-stage research. The first (simpler) method is based on determining the crack depth interval on the basis of many equations for each crack interval at 5 mm intervals. The method was verified by magneto-powder and ultrasonic tests and by drilling the disc in the middle of the fracture in order to measure it. The method allows to estimate the crack with an accuracy of approximately 85%.

In the second method, the surface fracture profile of the brake discs along their length was compared to the arc of a circle. This was proved by fitting the circle equation to the actual depth measurements made with a Karl Deutsch RMG 4015 potential probe.

Dependence of the depth from the crack length up to approx. 70 mm is linear, then it stabilizes and becomes independent of the increase in the crack length. Based on this relationship, it can be concluded that for cracks longer than 70 mm require additional tests for which it will be difficult to obtain a representative group of disks with such failures due to the relatively rare occurrence of such friction ring defects. The accuracy of the second method of crack depth estimation is 95%.

It should be noted that the method of propagation of surface cracks described in the article applies only to brake discs made of cast iron. In the case of steel discs, the course of the depth changes along the fracture length may be different.

Further directions of research on surface cracks should concern a detailed understanding of the phenomenon of hot spots in terms of its identification and modeling. Some studies have noted that this may be related to the shape of the friction material and its contact with the disc. Therefore, work on surface cracks should involve doing brake friction tests (hot-spots phenomenon), material tests and numerical analyses. Further understanding of these issues will have a direct impact on reducing the propagation of surface cracks in railway disc brakes.

## Figures and Tables

**Table 1:** Bench test plan.

Type of Test	Speed v (km/h)	Pad Pressure to the Brake Disc N (kN)	Braking Mass on the Brake Disc M (t)	Braking Deceleration a (m/s²)	Number of Brakes
A—local traffic	40, 80, 100, 120 and 140	55	7.5	0.7	72
B—constant braking power of 21, 25 and 34 kW	80	variable	6.3; 7.5; 10	-	9
C—fast driving	225	40, 55	4.4; 6.3; 7.5	0.9	18
D1—limit power	225	55	6	1; 1.1	140
D2—limit power	225	55	6	1; 1.1	70
				TOTAL	309

**Table 2:** Chemical composition in % of elements for cast iron discs used during the tests.

**Disc Made of Grey Cast Iron (T2) GJL-250 (GG-25)**							
C	Si	Mn	P	Cu	S	Ce	Sc
3.5	1.5	0.7	0.5	0.15	-	-	-
**Disc Made of Nodular Cast Iron (T1) GJS-400 (GGG-40)**							
C	Si	Mn	P	Cu	S	Ce	Sc
3.5	2.6	0.45	0.05	0.25	0.012	3.45	1.015

**Table 3:** Ranges of w_MC_ coefficients for different ranges of surface crack lengths.

No.	Crack Length Range in (mm)	Ratio Range w_MC_ = l_p_/g_p_
1	11–15	3.5–4.2
2	16–20	5.8–6.6
3	21–25	6.3–7.1
4	26–30	7.2–7.6
5	31–35	8.2–8.5
6	36–40	9.0–9.1
7	41–45	9.0–9.2
8	46–50	9.1–9.7
9	51–55	10.5–11.4

## Data Availability

The data presented in this study are available on request from the corresponding author.
